# A Novel Near-Infrared Fluorescence Probe THK-565 Enables *In Vivo* Detection of Amyloid Deposits in Alzheimer’s Disease Mouse Model

**DOI:** 10.1007/s11307-023-01843-4

**Published:** 2023-08-14

**Authors:** Fumito Naganuma, Daiki Murata, Marie Inoue, Yuri Maehori, Ryuichi Harada, Shozo Furumoto, Yukitsuka Kudo, Tadaho Nakamura, Nobuyuki Okamura

**Affiliations:** 1 Division of Pharmacology, Faculty of Medicine, Tohoku Medical and Pharmaceutical University, 1-15-1 Fukumuro, Miyagino-Ku, Sendai, Miyagi 983-8536 Japan; 2 Department of Pharmacology, Tohoku University Graduate School of Medicine, 2-1 Seiryo-Machi, Aoba-Ku, Sendai, Miyagi 980-8575 Japan; 3 Division of Radiopharmaceutical Chemistry, Cyclotron and Radioisotope Center, Tohoku University, 6-3 Aoba, Aramaki, Aoba-Ku, Sendai, Miyagi 980-8578 Japan; 4 Department of Aging Research and Geriatrics Medicine, Institute of Development, Aging and Cancer, Tohoku University, 4-1 Seiryo-Machi, Aoba-Ku, Sendai, Miyagi 980-8575 Japan

**Keywords:** Amyloid, Alzheimer’s disease, Imaging, Fluorescence

## Abstract

**Purpose:**

Noninvasive imaging of protein aggregates in the brain is critical for the early diagnosis, disease monitoring, and evaluation of the effectiveness of novel therapies for Alzheimer’s disease (AD). Near-infrared fluorescence (NIRF) imaging with specific probes is a promising technique for the *in vivo* detection of protein deposits without radiation exposure. Comprehensive screening of fluorescent compounds identified a novel compound, THK-565, for the *in vivo* imaging of amyloid-β (Aβ) deposits in the mouse brain. This study assessed whether THK-565 could detect amyloid-β deposits *in vivo* in the AD mouse model.

**Procedures:**

The fluorescent properties of THK-565 were evaluated in the presence and absence of Aβ fibrils. APP knock-in (APP-KI) mice were used as an animal model of AD. *In vivo* NIRF images were acquired after the intravenous administration of THK-565 and THK-265 in mice. The binding selectivity of THK-565 to Aβ was evaluated using brain slices obtained from these mouse models.

**Results:**

The fluorescence intensity of the THK-565 solution substantially increased by mixing with Aβ fibrils. The maximum emission wavelength of the complex of THK-565 and Aβ fibrils was 704 nm, which was within the optical window range. THK-565 selectively bound to amyloid deposits in brain sections of APP-KI mice After the intravenous administration of THK-565, the fluorescence signal in the head of APP-KI mice was significantly higher than that of wild-type mice and higher than that after administration of THK-265. *Ex vivo* analysis confirmed that the THK-565 signal corresponded to Aβ immunostaining in the brain sections of these mice.

**Conclusions:**

A novel NIRF probe, THK-565, enabled the *in vivo* detection of Aβ deposits in the brains of the AD mouse model, suggesting that NIRF imaging with THK-565 could non-invasively assess disease-specific pathology in AD.

## Introduction

Alzheimer’s disease (AD) is the most common cause of dementia in the elderly. Neuropathological hallmarks of AD include senile plaques (SPs) and neurofibrillary tangles (NFTs), primarily composed of amyloid-β (Aβ) and hyperphosphorylated tau proteins, respectively. The progressive accumulation of these proteins is considered a critical event in the pathogenesis of AD, leading to the development of anti-amyloid and anti-tau drugs aimed at preventing their buildup. To successfully develop anti-Aβ and anti-tau drugs for AD, it is essential to establish a method for non-invasive monitoring of Aβ and tau accumulation in the living brain [1].

Positron emission tomography (PET) imaging is currently the most practical modality for *in vivo* imaging of Aβ and tau in the human brain [2]. However, PET imaging requires facilities to generate positron-emitting radionuclides and synthesize radiolabeled tracers. Therefore, there has been increasing interest in the development of alternative techniques for the *in vivo* imaging of amyloid and tau deposits in the brain. Near-infrared fluorescence (NIRF) imaging with specific probes is considered a promising technique for *in vivo* imaging of Aβ and tau deposits in the brain because NIRF light can penetrate deeper tissues owing to minimal photon absorbance [3]. A variety of NIRF probes, including derivatives of Boron dipyrromethane (BODIPY), curcumin, and N,O-benzamide difluoroboron, have been developed for the *in vivo* detection of Aβ plaques and oligomers in the brain [4–12]. We previously developed a NIRF imaging probe named THK-265, which demonstrated the *in vivo* imaging of Aβ plaques in a mouse model of AD [13, 14]. However, insufficient fluorescence of THK-265 limits the sensitive detection of Aβ deposits in the brain. Here, we introduce a novel 3,4,10,10a-tetrahydropyrimido[1,2-a]indol-2(1H)-one derivative, THK-565, as an NIRF probe for amyloids. This compound allows the sensitive detection of amyloid deposits in the brains of AD mouse models.

## Materials and Methods

### Measurement of Fluorescence Properties of THK-565

**Fig. 1:**
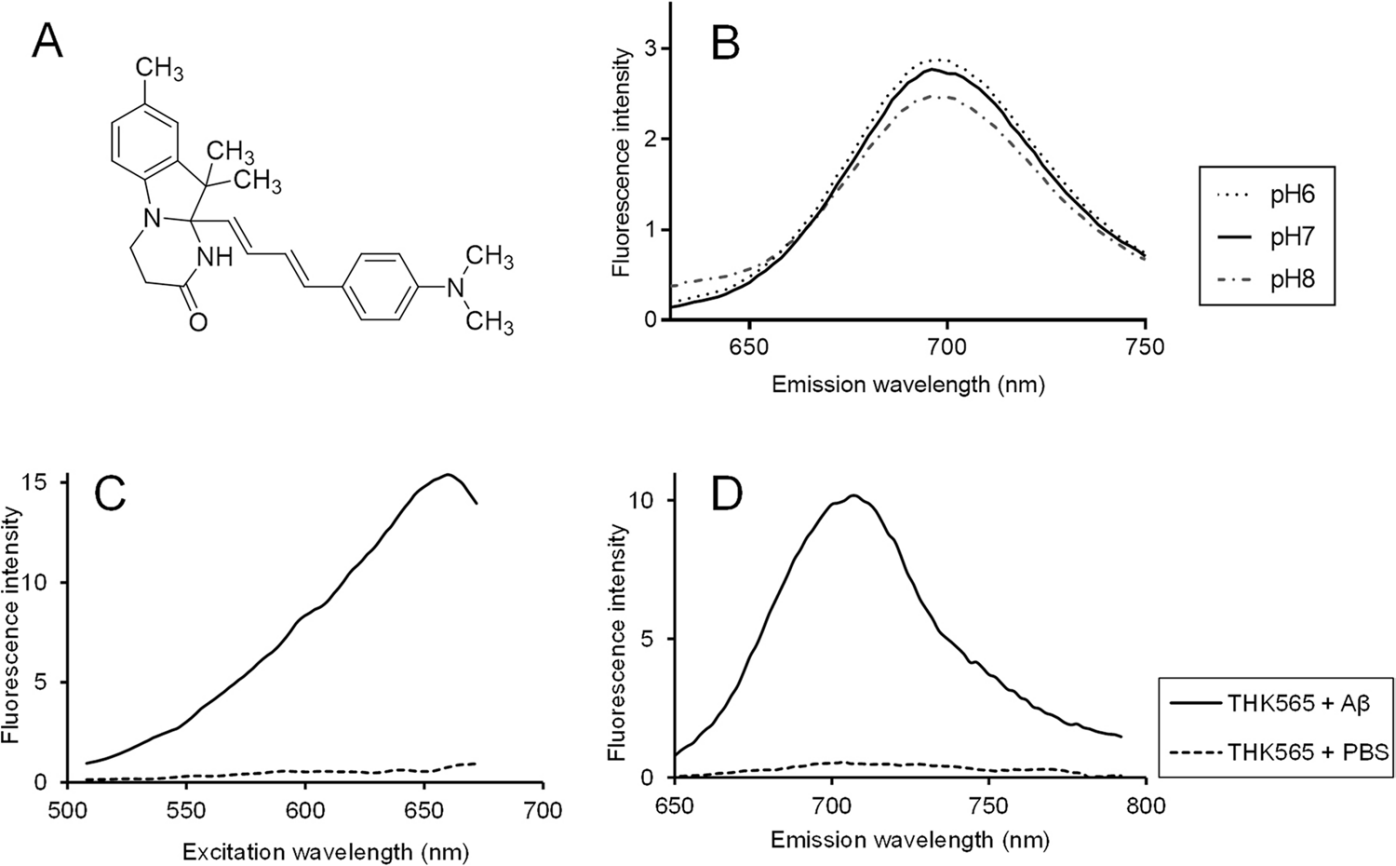
**A** Chemical structure of THK-565. **B** Fluorescence spectra of THK-565 under different pH conditions. **C**, **D** Excitation **C** and emission **D** spectra of THK-565 in PBS with and without Aβ fibril

The 10a-[(1*E*,3*E*)-4-(4-dimethylamino-phenyl)-buta-1,3-dienyl]-8,10,10-trimethyl-3,4,10,10a-tetrahydro-1H-pyrimido[1,2-a]indol-2-one (THK-565) (Fig. 1) was purchased from LaboTest (Halsbrücke, Germany). For Aβ fibril preparation, 20 μM of Aβ1-40 (Peptide Institute, Osaka, Japan) in 50 mM of potassium phosphate buffer was incubated at 37 °C on a NR-3 rotary shaker (Taitec, Koshigaya, Japan) over 4 days. Before fluorometric measurement, Aβ fibril solution was sonicated for 15 min at 45 kHz using a VS-100III ultrasonic cleaner (Iuchi, Osaka, Japan). THK-565 was dissolved in 50 mM potassium phosphate buffer (pH 7.4) with dimethyl sulfoxide (DMSO), and used for fluorescence measurements. The excitation and emission spectra of the complex formed between THK-565 and Aβ fibrils were measured using Varioskan LUX multimode microplate reader (Thermo Fisher Scientific, Waltham, MA, USA) and FP-6300-WRE-362 spectrofluorometer (JASCO, Tokyo, Japan). To measure the optimal excitation and emission wavelength of THK-565, the spectra of THK-565 at 1 μM concentration in a quartz cuvette were recorded with and without 5 μM aggregated Aβ1-40 in 50 mM of potassium phosphate buffer. The pH stability of THK-565 was evaluated by measuring the fluorescence spectra of THK-565 in Britton-Robinson buffer (1 μM final concentration) under different pH conditions (pH 6–8).

Absorption coefficient and quantum yield of THK-565 were measured using an absolute photo-luminescence (PL) quantum yield measurement system (C9920-02, Hamamatsu Photonics, Hamamatsu, Japan). The absorbed and emitted photon counts of a solution of 8 μM of THK-565 with aggregated Aβ1-40 in 50 mM of potassium phosphate buffer were measured and then used to calculate the PL quantum yields, expressed as the ratio of the number of photons emitted to the number of photons absorbed. Molar absorption coefficient was obtained from the equation ε = A / cl (A: the actual absorbance of light; c: molar concentration of THK-565 solution; l: the path length [cm]).

### *In Vitro* Fluorescence Binding Assay

Fluorometric analysis of THK-565 binding with Aβ fibrils was performed as described previously [13]. A total of 50 μM of Aβ1-40 in 50 mM of phosphate buffered saline (pH 7.4) was incubated at 37 °C on a Shake-XR shaker (Taitec, Koshigaya, Japan) over 4 days. Fluorescence spectra for a mixture containing 10 μM Aβ fibril and varying concentrations of THK-565 (3, 10, 30, 100, 300 nM, 1 and 3 μM final concentrations) were measured using a Varioskan LUX multimode microplate reader (Thermo Fisher Scientific, Waltham, MA, USA) at an excitation wavelength of 650 nm and an emission wavelength range of 670–780 nm. Fluorescence spectra for the same concentrations of THK-565 were additionally measured without mixture of Aβ fibril or with mixture of soluble Aβ. Spectra of the difference in fluorescence intensity (ΔF) between the mixture of Aβ fibrils and soluble Aβ were calculated for each THK-565 concentration. The maximum ΔF was used as an index of specific binding between THK-565 and Aβ fibrils. The binding constant (Kd) of THK-565 to Aβ fibril was calculated from a plot of THK-565 concentration versus ΔF of THK-565 at 700 nm emission wavelength, using GraphPad Prism software Version 9 (GraphPad, San Diego, CA).

### Determination of LogP Values

LogP value of THK-565 was determined by the high-performance liquid chromatography (HPLC) method according to the Organization for Economic Co-operation and Development guidelines with slight modifications. Briefly, 12 reference compounds with Log P values ranging between 0.5 and 4.0 were analyzed by HPLC under the following conditions: HPLC, a JASCO LC-2000 Plus series (JASCO); column, Inertsil® ODS-4 (4.6 × 150 mm, 5 µm; GL Sciences, Inc., Tokyo, Japan); mobile phase, 20 mM sodium phosphate buffer (pH 7.4)/acetonitrile (55/45); flow rate, 1.5 mL/min; UV absorbance, 254 nm; column temperature: 40 °C. A calibration curve of Log (tR-t0) (tR: retention time; t0: dead time) versus Log P was then created for each reference compound (R^2^ = 0.9469). THK-565 was also analyzed using the same HPLC method to measure the Log (tR-t0) value, which was then used for the determination of the Log P value from the calibration curve.

### Measurement of Brain Uptake of THK-565 in Normal Mice

All animal care and use protocols were conducted in accordance with the Standards of Humane Care and Use of Laboratory Animals of Tohoku Medical and Pharmaceutical University, Sendai, Japan. All animal experiments were approved by the Tohoku Medical and Pharmaceutical University Animal Experiment Committee (registration number: 22011-cn). All experiments involving animals also complied with the Guidelines for the Proper Conduct of Animal Experiments of the Ministry of Education, Culture, Sports, Science and Technology, Japan, and the ARRIVE guidelines for reporting animal research. All efforts were made to reduce the number of animals used and minimize animal suffering.

Blood–brain barrier (BBB) permeability of THK-565 was examined in mice. THK-565 (0.3 mg/kg) dissolved in 10% DMSO was administered into the tail vein of male c57BL/6 mice (body weight, 25–30 g; n = 3) (Japan SLC, Hamamatsu, Japan). Indocyanine green (ICG) (Daiichi Sankyo, Tokyo, Japan) was used as a negative control because ICG does not cross the intact BBB [15]. At 2, 10, 30, and 60 min after the injection of THK-565 or ICG, the mice were euthanized, and their brains were extracted without perfusion. The fluorescence signal intensity was measured using an IVIS Lumina Series III imaging system (PerkinElmer, Waltham, MA, USA). Cy5 and ICG filter sets were used to acquire the fluorescence of THK-565 and ICG, respectively. Brain uptake of THK-565 in mice was analyzed using HPLC with a fluorescence detector, as described previously [13]. THK-565 (1 mg/kg), dissolved in 5% ethanol solution, was administered to the tail vein of male ICR mice (body weight, 25–30 g; n = 3) (Japan SLC, Hamamatsu, Japan). Two minutes after injection, the mice were euthanized, and their brains were extracted. Brain homogenates were centrifuged at 14,000 rpm for 10 min and the supernatant was used for extraction. The mobile phase consisted of 20 mM NaH_2_PO_4_ and acetonitrile in 7:3 ratio at a flow rate of 1.5 mL/min. An FS-8020 fluorescence detector (Tosoh, Tokyo, Japan) was used at excitation and emission wavelengths of 341/426 nm. The percentage of the injected dose per gram of tissue (%ID/g) was calculated by comparing the tissue count with the tissue weight.

### *In Vitro *and* Ex Vivo *Labeling of Aβ Deposits with THK-565

All animal experiments, including the use of genetically modified mice, were approved by the Tohoku Medical and Pharmaceutical University Animal Experiment Committee (registration number:22011-cn) and the Tohoku Medical and Pharmaceutical University Centre for Gene Research (registration number: 2022–45). Brain sections from amyloid-β precursor protein knock-in (APP-KI) (RBRC06344; RIKEN BRC, Tsukuba, Japan) and wild-type (Wt) mice were immersed in 100 μM of THK-565 solution for 10 min. Sections stained with THK-565 were then briefly dipped in water, rinsed in phosphate-buffered saline, and examined using a fluorescence microscope (BZ-X710; Keyence, Osaka, Japan) equipped with a Cy5 filter set (excitation, 590–650 nm; emission, 663–738 nm). The *in vivo* binding of THK-565 to Aβ deposits was also examined in APP-KI and Wt mice. THK-565 solution was administered into the tail vein at a dose of 0.3 mg/kg. Sixty minutes post injection of THK-565, the mice were euthanized, and their brains were extracted without perfusion. Frozen brain sections were prepared using a CM3050 cryostat (Leica, Waetzlar, Germany) and imaged using a fluorescence microscope with a Cy5 filter set. The same sections were immunostained using a monoclonal antibody against Aβ (82E1; IBL, Gunma, Japan). For Aβ immunostaining, sections were immersed in a M.O.M^®^ blocking reagent (M.O.M^®^ Kit, Vector Laboratories, Newark, CA, USA) for 30 min, then incubated with 82E1 (1:1,000) overnight at 4 °C. Next, sections were incubated with a secondary biotinylated anti-mouse IgG antibody (M.O.M^®^ Kit) and Fluorescein Avidin DCS (M.O.M^®^ Kit). Sections were mounted using ProLong^®^ Gold Antifade Mountant (Thermo Fisher Scientific).

### *In Vivo* NIRF Imaging of Aβ Deposits in Mice with THK-565

Male and female 11–12 month-old APP-KI mice, and age-matched Wt mice were used for the *in vivo* measurement of amyloid deposits. *In vivo* NIRF imaging was performed using the IVIS Lumina Series III imaging system. THK-265 was used as the reference compound. Fluorescence signals were measured and analyzed using the Living Image software. A Cy5 filter set was used to acquire the THK-565 and THK-265 fluorescence, *in vivo*. Identical illumination settings were used for all images, and the fluorescence emission was normalized to photons per second per centimeter squared per steradian (p/s/cm^2^/sr). First, the mice were anesthetized with three mixed anesthetic agents (0.3 mg/kg medetomidine; 4 mg/kg midazolam; 5 mg/kg butorphanol). Then, they were injected with THK-565 (0.3 mg/kg), THK-265 (0.3 mg/kg), or saline (as a vehicle) via the tail vein. Images were acquired for 60 min under isoflurane anesthesia. Statistical comparison of fluorescence measurements was performed using the Mann–Whitney U test, an analysis of variance, and a Tukey multiple comparisons test, for a significance level of *p* < 0.05.

## Results

### Fluorescence Properties of THK-565

**Fig. 2:**
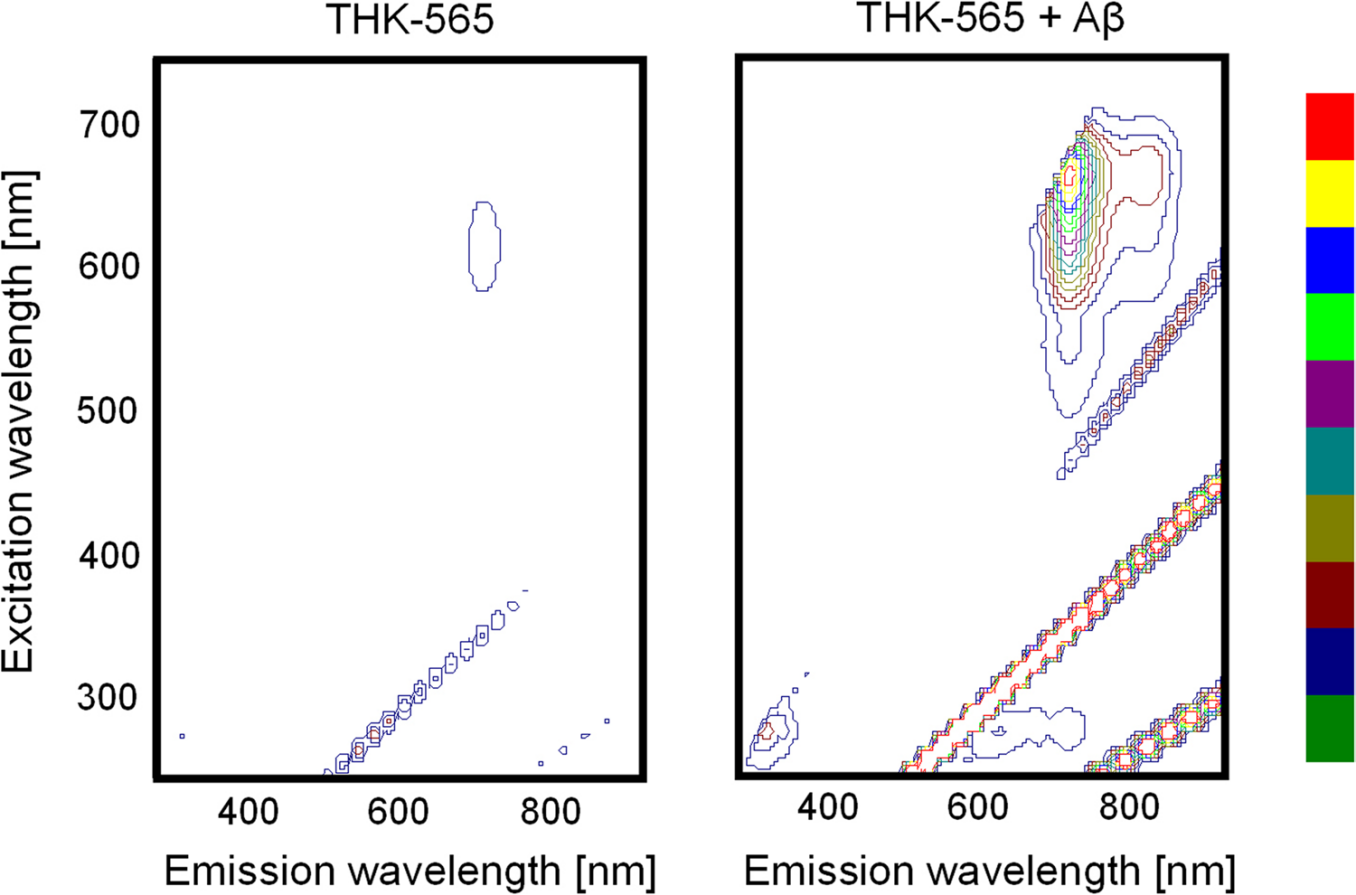
Fluorescence contour map of THK-565 solution and the THK-565/Aβ fibril complex

The fluorescence spectra of THK-565 in PBS were measured with and without Aβ fibrils (Figs. 1 and 2). The maximum excitation and emission wavelength of THK-565 were 620 nm and 694 nm in potassium phosphate buffer, respectively, while they were 650 nm and 704 nm in the presence of Aβ fibrils, respectively (Fig. 2). These values were within the range of the optical window, suggesting that the fluorescence signal of this compound can penetrate body tissues. The fluorescence spectra of THK-565 also showed a slight bathochromic shift after binding to Aβ fibrils. Furthermore, THK-565 exhibited high molar absorption coefficients (53,072 M^−1^ cm^−1^) and moderate quantum yield (7.1%) in Aβ1-40 solution. The fluorescence spectrum of THK-565 did not change significantly under different pH conditions (Fig. 1B).

### THK-565 Binding to Protein Fibrils

**Fig. 3:**
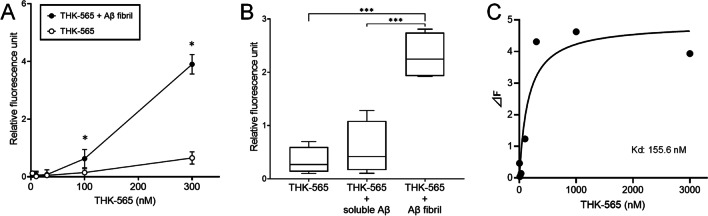
*In vitro* binding of THK-565 to Aβ fibrils. **A** Comparison of THK-565 fluorescence intensity and that of the THK-565/Aβ fibril complex at different concentrations. *p < 0.05, compared with the fluorescence intensity of THK-565 alone. **B** Comparison of fluorescence intensity of THK565 alone, THK-565 with soluble Aβ, and THK-565 with Aβ fibrils. **C** Saturation curve of THK-565 binding to Aβ fibrils. ***p < 0.05, compared with fluorescence intensity of THK-565 with Aβ fibrils

**Fig. 4:**
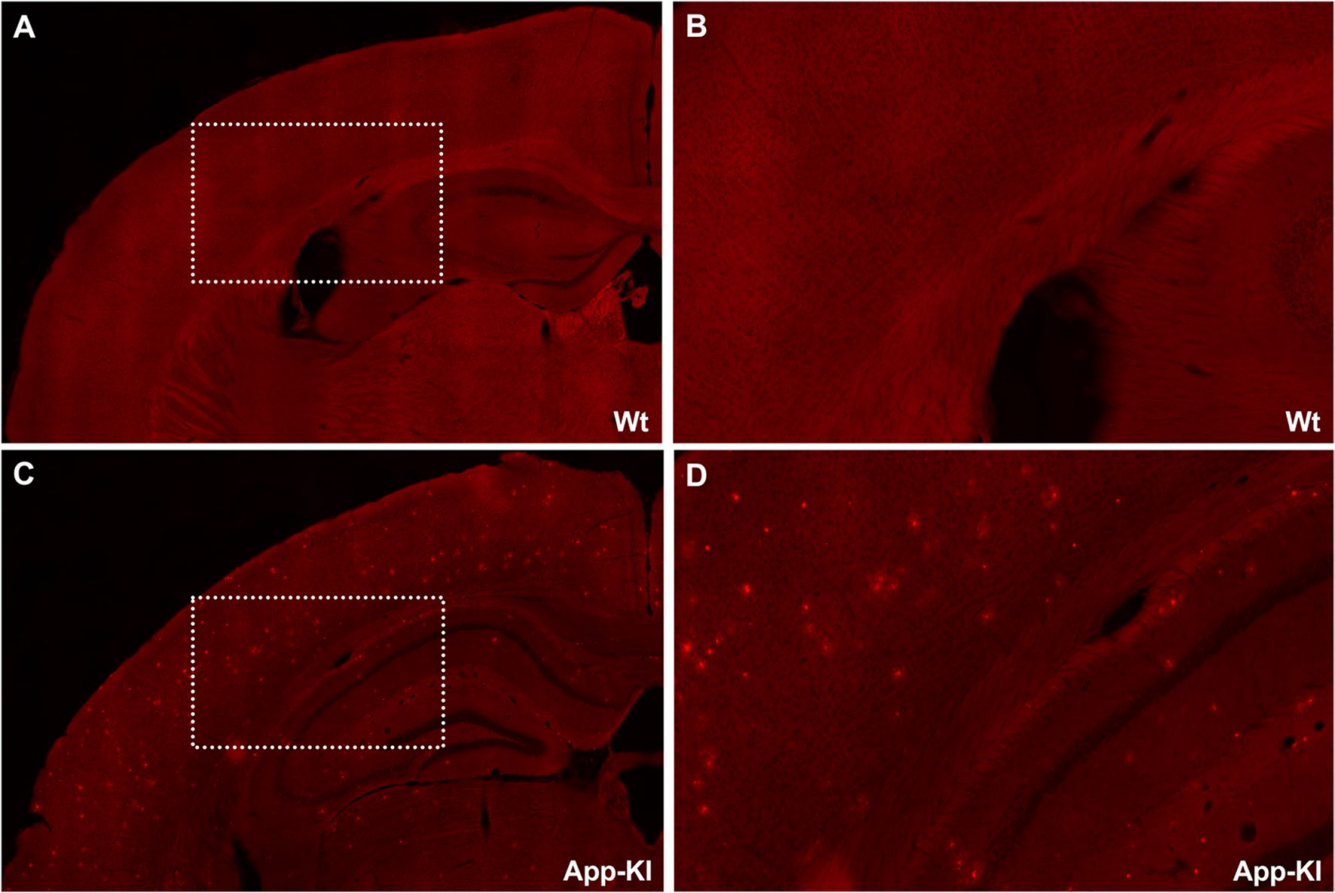
*In vitro* THK-565 binding to Aβ and tau deposits in the mouse models of AD. THK-565 staining of brains sections from wild-type (Wt) **A**, **B** and amyloid-β precursor protein knock-in (APP-KI) **C**, **D**

The fluorescence intensity of THK-565 was enhanced in a concentration-dependent manner upon mixing with Aβ fibrils (Fig. 3A). A significant increase in THK-565 fluorescence was observed at concentrations of 100 and 300 nM. In contrast, no significant increase in THK-565 fluorescence was observed after mixing with soluble Aβ peptide (Fig. 3B). These findings reflect the hyperchromic fluorescence shift of THK-565 upon binding to β-sheet structures. The ΔF value increased with increasing THK-565 concentration, and the calculated Kd value of THK-565 to Aβ was 155.6 nM (Fig. 3C). The *in vitro* binding ability of THK-565 to Aβ deposits was investigated in brain sections from APP-KI mice. THK-565 observably clearly stained the amyloid plaques in APP-KI mouse brain sections. In contrast, no significant THK-565 binding was detected in the brains of Wt mice (Fig. 4).

### LogP and BBB Permeability of THK-565

**Fig. 5:**
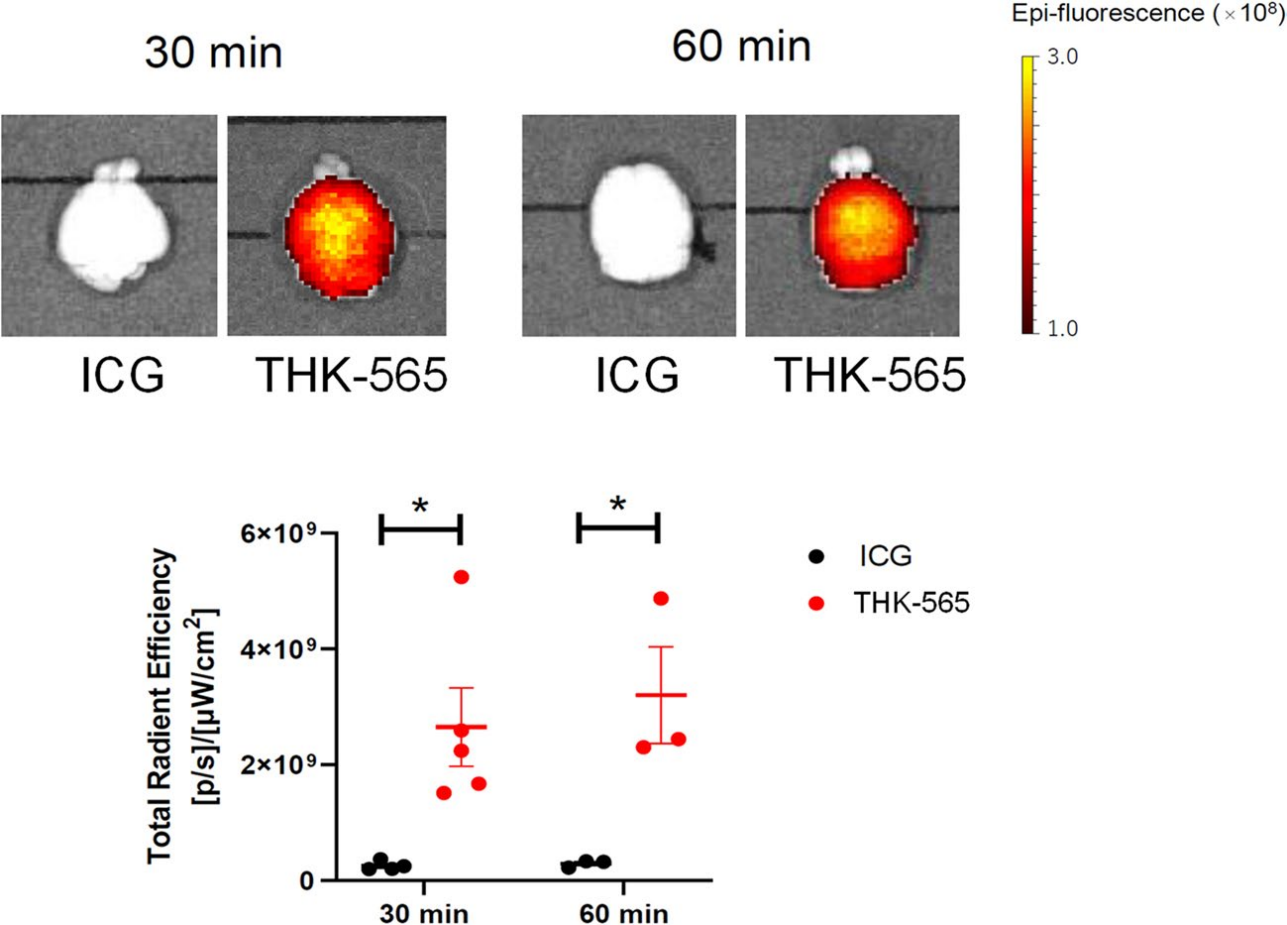
*Ex vivo* measurement of fluorescence intensity of brain tissue after 30 min and 60 min intravenous administration of THK-565 and ICG in mice *p < 0.05, unpaired t-test (30 min *p* = 0.017, 60 min *p* = 0.025)

To assess BBB permeability of THK-565, LogP values were determined using HPLC. The LogP value of THK-565 was 2.36, suggesting high BBB permeability of this compound. The BBB permeability of THK-565 was investigated in normal mice. ICG was used as a negative control in this analysis. As shown in Fig. 5, the fluorescence intensity in the brain was significantly elevated after the intravenous administration of THK-565, in contrast to the lack of remarkable elevation in the fluorescence signal in the brain after the administration of ICG. HPLC analysis of brain homogenates further demonstrated that the amount of brain THK-565 uptake was 0.95%ID/g at 2 min after intravenous injection in ICR mice.

### *In Vivo* Binding of THK-565 to Amyloid Deposits

**Fig. 6:**
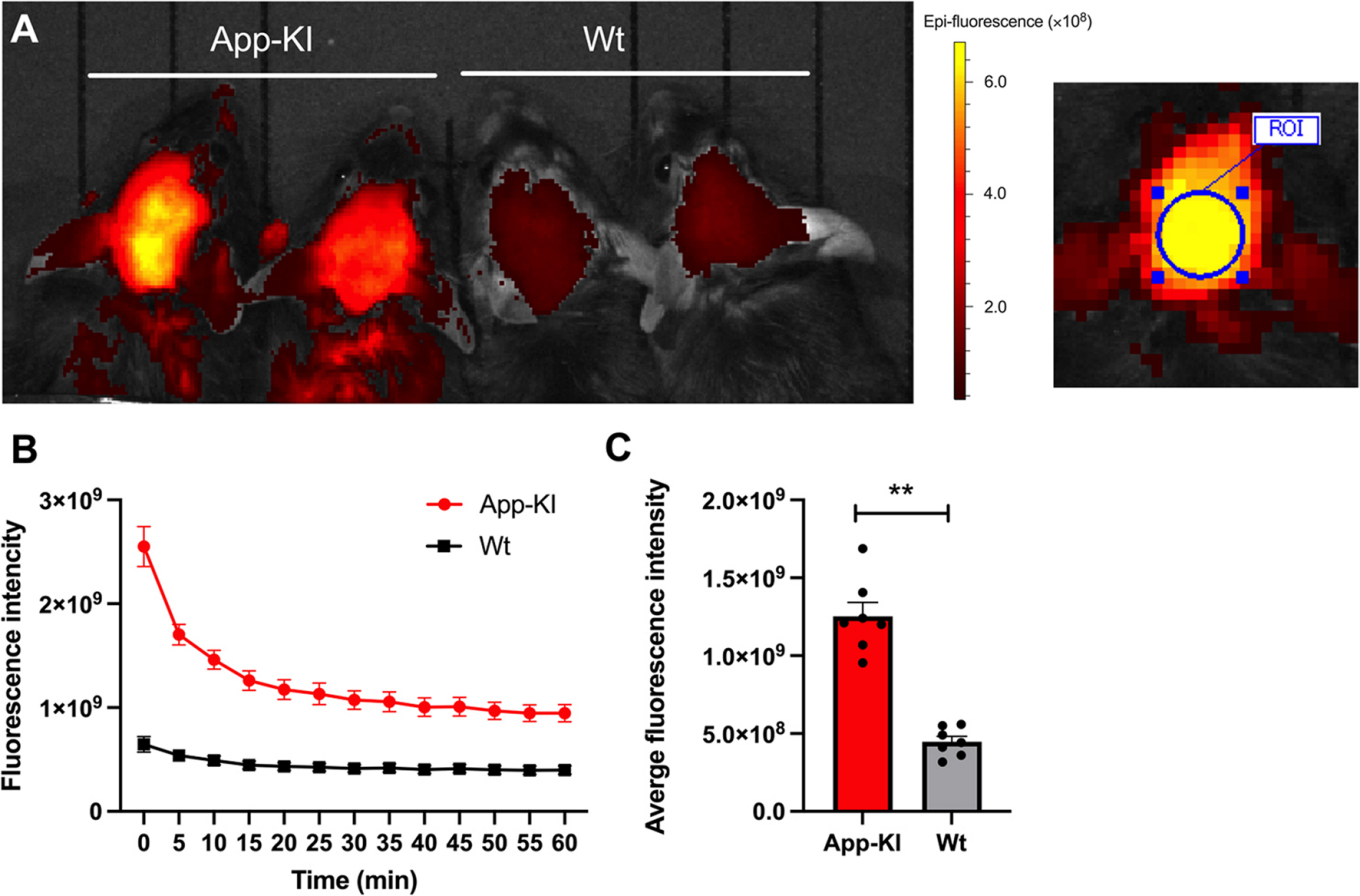
*In vivo* imaging of amyloid deposits using THK-565. **A** Images of the brains of 11–12 month-old amyloid-β precursor protein knock-in (APP-KI) and wild-type (Wt) mice acquired after intravenous administration of THK-565 and the placement of region of interest (ROI). **B** Fluorescence signal intensities of the heads of 11–12 month-old APP-KI and Wt mice as a function of time after the intravenous administration of THK-565. **C** Average fluorescence intensity of THK-565 between 0 and 60 min after injection in APP-KI and Wt mice

**Fig. 7:**
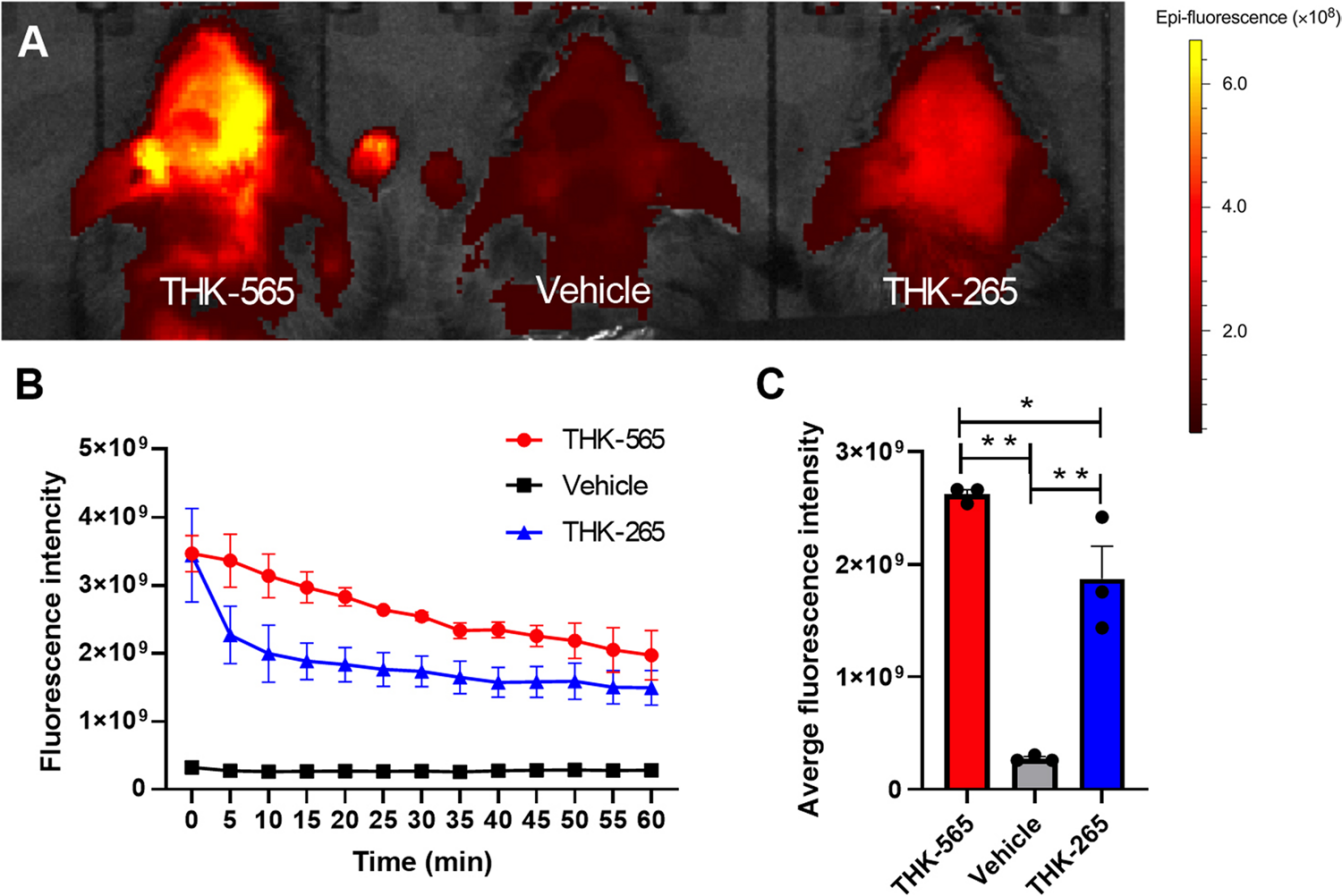
Comparison of *in vivo* fluorescence signals between compounds. **A** Images of the brains of 11–12 month-old amyloid-β precursor protein knock-in (APP-KI) mice acquired after intravenous administration of THK-565, THK-265, and vehicle. **B** Fluorescence signal intensities of the heads of 11–12 month-old APP-KI mice as a function of time after the intravenous administration of THK-565, THK-265, or vehicle. **C** Average fluorescence intensity of THK-565, THK-265, and vehicle between 0 and 60 min after injection in APP-KI mice.

**Fig. 8:**
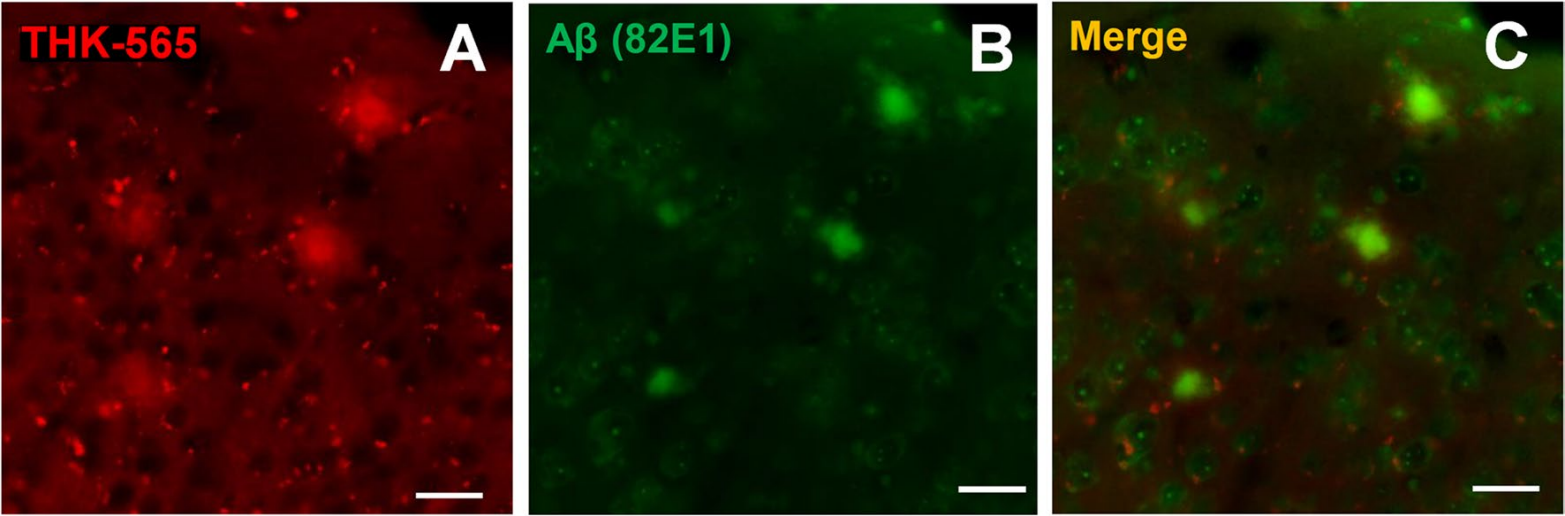
*Ex vivo* microscopic images. Images of brain sections from a 11–12 month-old amyloid-β precursor protein knock-in (APP-KI) mice **A** after intravenous administration of 0.3 mg/kg THK-565. **B** Aβimmunostaining in the same sections as (A). **C** Merged images of (A) and (B). Scale bar = 100 μm

Finally, NIRF imaging of the amyloid using THK-565 was performed in APP-KI mice. Following intravenous injection of 0.3 mg/kg THK-565, the fluorescence intensity of THK-565 rapidly increased in the heads of mice. To compare the fluorescence intensity of THK-565 treatment, a circular region of interest (ROI) (7.5 mm in diameter) was set on the heads (Fig. 6). The ROI was placed wider than the brain cortex to measure the fluorescence intensity, including scattered fluorescence. The fluorescence intensity of THK565 in the heads of APP-KI mice was consistently higher than that in the heads of Wt mice (Fig. 6). We further compared the *in vivo* fluorescence intensity of THK-565 with that of THK-265. In APP-KI mice, the fluorescence intensity of THK-565 was significantly higher than that of THK-265 (Fig. 7). *Ex vivo* fluorescence signals in the brain were measured to confirm whether the *in vivo* fluorescence signals in APP-KI mouse heads were derived from THK-565 fluorescence in the brain. The results demonstrated that the fluorescence signals in the brains of APP-KI mice were significantly stronger than those in Wt mice (data not shown). Further analysis using fluorescence microscopy confirmed that the THK-565 signals corresponded to immunostained Aβin the brain sections of APP-KI mice (Fig. 8), indicating that intravenously administered THK-565 could enter the brain and selectively bind to intracranial amyloid deposits.

## Discussion

Recent progress in molecular imaging has enabled non-invasive monitoring of amyloid and tau deposits in the living brain. PET imaging of amyloids has been widely used to evaluate the efficacy of anti-amyloid drugs [16]. In addition to Aβ specific probes, tau-specific PET probes have also been successfully developed and utilized in clinical practice [17]. However, PET imaging requires expensive devices such as PET scanners, cyclotrons, and radiosynthetic devices. NIRF imaging is expected to be an alternative *in vivo* technique for PET imaging [18]. The advantage of NIRF imaging over PET imaging is that it does not require expensive equipment such as cyclotrons and PET scanners. Another advantage is that NIRF imaging allows real-time data acquisition with excellent temporal resolution. In contrast to radioactive probes, fluorescent probes exhibit stability over extended time periods, facilitating the analysis of brain samples following *in vivo* experiments. As the fluorescence intensity of NIRF probes correlates well with the formation of protein fibrils, NIRF imaging can be used to evaluate the status of protein aggregates in the living brain. Although NIRF imaging has not yet been applied to human studies, it has recently been used for the *in vivo* monitoring of AD pathology in small animal models of AD [19–22].

Unlike visible light, NIRF can penetrate deeper tissues because of the minimal absorption coefficient of NIRF light via biological tissues and the minimization of tissue autofluorescence, which offers a higher target-to-background ratio [4]. There are several requirements for the sensitive detection of amyloids using NIRF imaging. The probe should have (1) high binding affinities to Aβ, (2) exceptional blood–brain barrier permeability (logP values between 2 and 3.5), (3) rapid clearance of unbound probe from normal brain tissue, and (4) acceptable fluorescence properties (excitation and emission wavelengths in the NIR range of 650–900 nm). Ideally, NIRF probes for amyloid aggregates should show an increase in fluorescence intensity upon binding to protein fibrils and a shift in fluorescence wavelength so that they can be distinguished from unbound probes.

Various NIRF probes have been reported for *in vivo* imaging of Aβ plaques in the brain [4]. AOI-987 has absorption and emission wavelengths within the NIR range, penetrates the BBB after intravenous injection, and specifically labels Aβ plaques in the brain [5]. However, small Stokes shifts (25 nm), marginal fluorescence changes upon mixing with Aβ, and slow clearance rates in the brain limit the sensitive detection of Aβ deposits in the brain [23]. NIAD-4 was also developed as an NIRF probe for Aβ deposits [7]. NIAD-4 showed excellent binding affinity for Aβ fibrils (*K*i = 10 nmol/L) and significantly enhanced fluorescence intensity when mixed with Aβ fibrils. However, the maximum emission wavelength of NIAD-4 is 603 nm, falling outside the optimal range for NIRF probes. Curcumin derivatives have also been explored for imaging Aβ fibrils [24]. One such derivative, CRANAD-2, demonstrated high affinity for Aβ fibrils and substantial alterations in fluorescence intensity and wavelength upon binding to Aβ fibrils. After intravenous administration, CRANAD-2 effectively distinguished between APP transgenic and Wt mice. BODIPY-based probes have been designed for in imaging amyloid in the brain [25, 26]. These derivatives showed high affinity and specificity to Aβ aggregates and clearly stained amyloid plaques in brain sections of APP transgenic mice. In addition, *ex vivo* fluorescent staining of brain sections displayed selective binding of amyloid plaques with little nonspecific binding. Verwilst et al. reported novel Tau-1 and Tau-2 for imaging tau aggregates in the brain [27]. These compounds showed large Stokes shift and emission in the NIRF range. Tau-1 demonstrated efficient BBB penetrability and demonstrated a distinct selectivity for tau tangles over Aβ plaques, as well as its capability for *in vivo* imaging in a transgenic mouse model [27]. Preclinical studies using these probes demonstrated the usefulness of NIRF imaging in AD mouse models.

We screened NIRF compounds for *in vivo* imaging of amyloid deposits and identified THK-265 as a candidate NIRF probe [13]. In the present study, the fluorescence signal after the intravenous administration of THK-565 was greater than that after THK-265 administration in the brains of APP-KI mice. A previous study demonstrated that THK-265 detected amyloid deposits in the brains of APP transgenic mice, and that the *in vivo* fluorescence intensity of THK-265 correlated well with the amyloid plaque burden [13]. However, THK-265 displayed inadequate brain uptake (0.04%ID/g at 2 min post injection) in mice. Moreover, the maximum excitation and emission wavelengths of the THK-265 were 630 nm and 650 nm, respectively, which are within the optimal range for NIRF probes. Compared to THK-265, THK-565 showed higher brain uptake (0.95%ID/g at 2 min post injection) in mice. The maximum excitation and emission wavelengths of THK-565 were within the optical window (> 650 nm). These wavelengths were longer than those of THK-265 and AOI-987. Furthermore, both hyperchromic and bathochromic effects of THK-565 were observed upon the binding to Aβ fibrils. Fluorescence intensity of THK-565 enhanced when THK-565 bound to Aβ fibrils in a nanomolar concentration range, indicating that THK-565 has excellent fluorescence properties as an NIRF probe. Given that THK-565 has a unique structural framework not observed in other fluorescent probes, it could potentially be the cause of bathochromic shift. However, considering the subtle 10 nm shift, it remains a challenge to determine whether this change is a result of environmental modifications due to THK-565 binding to the hydrophobic pockets of amyloid aggregates, or if it is a distinctive shift tied to the chemical structure (skeleton) of THK-565.

NIRF imaging with THK-565 holds the potential for enabling the quantitative assessment of Aβ aggregates in the living brain. In the future, we aim to conduct a longitudinal study to verify whether the severity of Aβ pathology can be evaluated using this probe. It is also crucial to investigate whether THK-565 can noninvasively detect protein aggregates other than Aβ, as THK-565 is expected to possess a high affinity for various protein fibrils that form β-sheet structures.

## Conclusions

We successfully developed a novel NIRF probe, THK-565, for evaluating Aβ burden in AD mouse models. THK-565 demonstrated outstanding fluorescence and pharmacokinetic properties as an NIRF agent. Using the NIRF imaging device, Aβ aggregates in the brains of AD mouse models were detected after the intravenous administration of THK-565. Based on these results, we conclude that THK-565 serves as a promising NIRF probe for the noninvasive detection and monitoring of AD pathology.

## Data Availability

The data that support the findings of this study are available from the corresponding author upon reasonable request.
